# The Impact of Genotoxicity in Coke Oven Workers: Systematic Review With Meta‐Analysis

**DOI:** 10.1002/jat.70098

**Published:** 2026-02-16

**Authors:** Thiago Guedes Pinto, Vinícius Fialho do Nascimento, Giovanna Abreu Holanda Guerra, Wilton Mitsunari Takeshita, Raquel Alves Sales, Andrea Cristina de Moraes Malinverni, Luciana Lopes Guimaraes, Daniel Araki Ribeiro

**Affiliations:** ^1^ Department of Biosciences, Institute of Health and Society Federal University of São Paulo, UNIFESP Santos São Paulo Brazil; ^2^ Department of Pathology, Laboratory of Molecular and Experimental Pathology Federal University of São Paulo, UNIFESP São Paulo São Paulo Brazil; ^3^ Department of Diagnosis and Surgery, School of Dentistry São Paulo State University (UNESP) Araçatuba São Paulo Brazil; ^4^ Santa Cecilia University Santos São Paulo Brazil

**Keywords:** coke oven workers, genotoxicity, polycyclic aromatic hydrocarbons

## Abstract

This systematic review (SR) with meta‐analysis investigates the genotoxicity potential of coke oven workers (COWs) exposed to polycyclic aromatic hydrocarbons (PAHs) through a comprehensive analysis of studies retrieved from PubMed, SCOPUS, and Web of Science. The comparisons were defined as standardized mean difference (SMD), and 95% confidence intervals (CIs) were established. A systematic search conducted in May 2025 identified 21 relevant studies, which employed different assays, such as the micronucleus assay (MA) and the comet assay (CA) in order to assess DNA damage. The outcomes suggested that 21 of the reviewed studies observed genotoxic effects related to this exposure, with 20 inducing micronucleus formation and chromosomal abnormalities. As for the quality assessment, a total of 18 studies were classified as Strong, and three (out of 21) were deemed as Moderate. No study was categorized as Weak, which proves our findings can be considered trustworthy. The meta‐analysis (six studies) revealed a statistically significant difference between COWs and the control group, for both the MN (SMD = 0.70, 95% CI, 0.26–1.15, *p* = 0.002) and CA (SMD = 0.86, 95% CI, 0.34–1.38, *p* = 0.001) with high heterogeneity. We concluded that there is a potential for genotoxicity in COWs. This certainly shows the importance of further investigation and regulatory oversight to ensure coke oven professionals' safety. Also, we understand such findings are vital for clarifying the role of biomarkers related to genotoxicity due to this occupational exposure.

## Abstract 2

Coke oven workers are in an occupational group at high risk for genotoxicity, as determined by cytogenetic, cellular, and molecular endpoints. This finding certainly shows the importance of regulatory oversight to ensure the safety of these professionals, since genetic damage is the initial step in some chronic degenerative diseases, such as cancer.

## 1 Introduction

Coke oven emissions (COEs) are complex mixtures of pollutants, prominently featuring polycyclic aromatic hydrocarbons (PAHs) and nitro‐PAHs, both known for their genotoxic and carcinogenic properties (Li et al. 2015; Vimercati et al. 2020). The chemical composition of these emissions varies with the coke production technology employed (IARC 2012). Long‐term occupational exposure to PAHs among coke oven workers (COWs) has been linked to elevated risks of cancers of the lung, bladder, stomach, and skin (Samir et al. 2019). In particular, a 30‐year follow‐up study confirmed increased mortality from respiratory system cancers and prostate cancer among COWs.

Genotoxicity plays a critical role in occupational exposure, since genetic damage is involved in the initial phase of chemical carcinogenesis. Particularly, cytogenetic analysis of peripheral blood lymphocytes by means of micronucleus assay is a well‐established method for assessing occupational exposure to clastogenic agents such as PAHs (Fenech 2000). Other cytogenetic endpoints include chromosomal aberrations (CAs), sister chromatid exchanges (SCEs), and DNA strand breaks, all of which serve as biomarkers of genotoxic effects (Bonassi et al. 2007). In fact, some authors have shown a correlation between increased genetic damage and higher cancer incidence (Bonassi et al. 2008). On the other hand, some biomarkers such as urinary 1‐hydroxypyrene (1‐OHP) and 8‐hydroxy‐2′‐deoxyguanosine (8‐OHdG) have also been utilized to monitor internal PAH exposure and subsequent oxidative DNA damage (Hong et al. 2000). The results have demonstrated a significant correlation between these two biomarkers among COWs, indicating a link between PAH exposure and DNA oxidation (Wu et al. 2004).

Given the great relevance of genotoxicity following occupational exposure under different contexts and paradigms, the current study aims to systematically review the literature to determine whether occupational exposure to PAHs among COWs is consistently associated with genotoxicity.

## 2 Material and Methods

Studies were included if they met all the following criteria: (1) original observational studies evaluating cytogenetic/genotoxic outcomes (e.g., CAs, SCE, micronuclei) in relation to occupational PAH exposure; (2) studies assessing different polymorphisms in modulating genotoxic effects; (3) studies conducted in human populations, specifically workers in coke oven or similar industrial settings; (4) manuscripts published in peer‐reviewed journals and written in English; (5) availability of sufficient methodological detail and extractable data on outcomes and exposures.

Exclusion criteria were (1) reviews, conference abstracts, editorials, case reports, or letters; (2) experimental studies in vitro or in non‐human models; (3) studies not reporting any genotoxic or cytogenetic outcomes; (4) lack of clear exposure characterization or absence of a comparator group; (5) full texts not accessible or not published in English; (6) insufficient data for extraction or quality assessment.

## 3 Search Strategy

A systematic search of the literature was performed in May 2025 across the databases PubMed, Scopus, and Web of Science. The search strategy included the combination of PubMed, SCOPUS, and Web of Science through the following Boolean operators: (“Polycyclic aromatic hydrocarbons” OR “PAHs” OR “coke oven emissions”) AND (“Genotoxicity” OR “Cytogenetic damage” OR “Chromosomal aberrations” OR “Sister chromatid exchange” OR “Micronuclei”) AND (“polymorphisms” OR “polymorphism”) AND (“Occupational exposure” OR “Workers”).

No restrictions were applied regarding the publication date to capture both historical and recent evidence. Additionally, the reference lists of all selected manuscripts were hand‐searched to identify potentially eligible studies not captured in the initial database queries.

## 4 Study Selection and Data Extraction

Five independent reviewers screened the titles and abstracts for eligibility. Full texts of potentially relevant studies were then assessed based on the inclusion criteria. Although no significant disagreement came up, divergences among reviewers were resolved through discussion and consensus. For each included study, the following data were extracted: study design, population characteristics, exposure assessment method, type of cytogenetic/genotoxic assay used, polymorphism genotypes, presence of a control group, blind analysis, proper statistical analysis, and main findings.

The detailed search strategy and screening process are summarized in Table 1.

**TABLE 1:** Search strategy.

Electronic databases used	Search strategy (August 2025)
PubMed https://www.ncbi.nlm.nih.gov/pubmed/ Scopus https://www.scopus.com Web of Science https://www.webofscience.com/wos/alldb/basic‐search	(polycyclic aromatic hydrocarbons) OR (coke oven emissions) AND (DNA Damages) OR (Damage, DNA) OR (Damages, DNA) OR (DNA Injury) OR (DNA Injuries) OR (Injuries, DNA) OR (Injury, DNA) OR (Genotoxic Stress) OR (Genotoxic Stresses) OR (Stresses, Genotoxic) OR (Genotoxicity) OR (Mutagenicity) OR (comet assay) OR (micronucleus assay) OR (sister chromatid exchange) OR (chromosomal aberration test) OR (Stresses, Genotoxic) (micronucleus) OR (micronucleated cell) OR (chromosome damage) OR (chromosomal injury) OR (chromosome breakage) OR (chromosome aberration test) OR (genetic polymorphism) OR (gene polymorphism) OR (polymorphism)) OR (polymorphic gene) OR (gene polymorphic) OR (gene, polymorphism) OR (gene, polymorphic).AND (mammalian) OR (occupational) OR (worker) OR (job).

## 5 Data Extraction and Quality Assessment

Data from the eligible studies were independently extracted by two reviewers using a standardized protocol tailored to the objectives of this review. The collected information included: authorship, publication year, country of study, characteristics of the study population (such as occupational role, age distribution, smoking habits), exposure parameters (e.g., environmental PAH levels, 1‐OHP concentrations), cytogenetic/genotoxicity endpoints assessed (including CAs, SCEs, and micronuclei), number of analyzed cells, type of assay performed, inclusion of genetic polymorphism data (including various detoxification, metabolic, and DNA repair gene variants), blinding status of outcome evaluation, statistical approaches employed, presence of negative or low‐exposure control groups, and main findings related to genotoxic outcomes and genetic susceptibility. This methodology has been broadly used by our research group (Guedes Pinto et al. 2024; Pinto et al. 2025).

In parallel with data extraction, a methodological quality and risk of bias assessment was carried out for each included study. This evaluation followed an adapted framework based on checklists used in prior systematic reviews in occupational and environmental toxicology. Key domains assessed included: clear definition of exposure and outcome measures, adjustment for relevant confounders (such as age, smoking, alcohol consumption), implementation of blinding during outcome assessment, sample size adequacy, completeness of genotyping data, and appropriateness of statistical analyses.

Based on these domains, each study was assigned a quality grade. Studies that met all criteria without critical limitations were rated as Strong. Studies that lacked control of more than one methodological factor were classified as Moderate. Studies with two or more uncontrolled confounding factors, poor exposure definition, or other significant biases were rated as Weak. Final classifications were reached by consensus among reviewers after independent assessment (Guedes Pinto et al. 2024; Pinto et al. 2025).

## 6 Meta‐Analysis

The meta‐analysis using a random‐effects model was performed to compare COWs and control. The random‐effects model with the Der Simonian‐Laird method was used to minimize the influence of heterogeneity on the articles included. The standardized mean difference (SMD) was used as the effect measure and it was calculated by the means and standard deviations (SDs) from COWs and control. The effect size was determined by calculating Cohen's *d* statistics. A value effect of 0.2, 0.5, and 0.8 was considered a small, medium, and large effect, respectively (Malacarne et al. 2023; Pinto et al. 2025).

Forest plots were used to display graphically the effect sizes and 95% confidence intervals (CIs). A two‐tailed *p* < 0.05 was used to determine the statistical significance. The heterogeneity among the studies included was analyzed with Cochran's Q test and quantified with *I* ^2^ statistics (Malacarne et al. 2023; Pinto et al. 2025). Indices lower than 25% indicated low heterogeneity among the studies, between 25% and 75% moderate, and above 75% high. The analyses were performed using the RevMan software Version 5.4.1 (The Cochrane Collaboration, Oxford, UK) (Malacarne et al. 2023; Pinto et al. 2025).

## 7 Results

### 7.1 Study Selection

**FIGURE 1:**
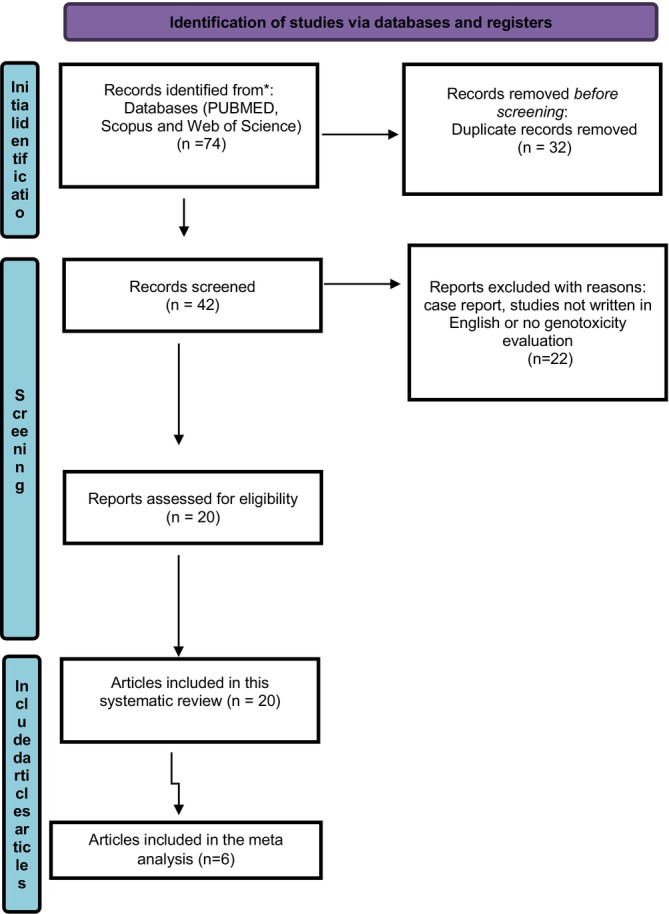
The flow chart of the study.

Our initial search of relevant literature identified 74 studies related to COWs; however, 32 of these were duplicates and were excluded from further review. After evaluating the titles and abstracts, 21 studies were found to be unrelated to the focus of this review and were subsequently discarded. This exclusion criterion included reviews, case reports, commentaries, editorials, non‐English publications, and letters to the editor. The full texts of 21 studies were carefully reviewed by the authors (Figure 1).

### 7.2 General Characteristics of the Included Studies

The most important characteristics of the evaluated studies can be visualized in Table 2. A total of 21 studies were evaluated, 13 being conducted in China, two in Taiwan, and one in each of the following countries: Egypt, India, Turkey, Germany, Netherlands, and Slovakia. Different exposure times were reported and duly presented in Table 2.

**TABLE 2:** The most important characteristics of the studies included in the systematic review.

Author	Year of publication	Country	Exposure period/time
Samir et al.	2019	Egypt	≥ 1 year
Xiaoliang et al.	2015	China	> 1 year
Dai et al.	2014	China	> 1 year
Sureshkumar et al.	2013	India	0–12 years
Ada et al.	2013	Turkey	≥ 3 months
Huang et al.	2012	China	> 6.4 years
Chen et al.	2011	Taiwan	≥ 1 year
Wang et al.	2010	China	> 1 year
Cheng et al.	2009	China	> 6 months
Yang et al.	2009	China	> 6 months
Yang et al.	2008	China	> 6 months
Qiu et al.	2007	China	Not reported
Cheng et al.	2007	China	> 1.5 months
Cheng et al.	2007	China	Not reported
Chen et al.	2006	China	> 6 months
Leng et al.	2005	China	> 1.5 months
Leng et al.	2004	China	> 6 months
Wu et al.	2004	Taiwan	≥ 1 year
Marczynski et al.	2002	Germany	≥ 1 year
van Delft et al.	2001	Netherlands	≥ 1 year
Kalina et al.	1998	Slovakia	≥ 5 years

### 7.3 Variables Related to Occupation Reported and PAHs

All included studies performed some verification of genotoxic outcomes induced by coke oven following occupational exposure. All studies (11 out of 21) performed the micronucleus assay using peripheral blood or buccal mucosa cells and some performed other analyses outside the scope of this review. Between the studies carried out, only 8 out of 21 performed comet assay to evaluate genotoxic damage. Among them, Marczynski et al. (2002), van Delft et al. (2001), Kalina et al. (1998), and Wang et al. (2010) used peripheral blood, while van Delft et al. (2001) also included urothelial cells in their analysis (Table 3).

**TABLE 3:** Describes the variables related to occupational exposure of polycyclic aromatic hydrocarbons and mutagenicity. First, all studies presented control groups for proper comparison.

Author	Cell type	*N*	Gender	Age	Assays	*N* of evaluated units	Stain	Evaluated parameters	Inclusion criteria	Cytotoxicity analysis	Blind analysis	Proper statistical description	Negative control	Positive control
Samir et al.	Peripheral blood lymphocytes	W: 85 C: 85	Male	W: 45.6 years C: 44.9 years	HPLC qPCR ELISA Gas chromatography	Not specified	Not specified	Urinary 1‐OHP 8‐OHdG urinary Gene expression CYP2E1 BPDE‐DNA adduct Genotyped for XRCC1 Arg/GIn Air sampling and analysis of airborne PAHs Demographic and lifestyle factors	Yes	No	Yes	ANOVA Kruskal–Wallis Spearman correlation	Yes	No
Xiaoliang et al.	Peripheral blood	W: 922 C1: 1.017 cancer + non‐cancer (2.034) C2: 617 cancer 661 non‐cancer (1.278)	Male	W: 42.43 C1: 60.3; 59.7 C2: 60.3; 61.3	MN ELISA SNP	1000	Giemsa	Cell count Plasma concentration of BPDE‐Alb Genotype frequencies of APEX1: rs1760944	Yes	No	No	Komogorov–Smirnov Kruskal–Wallis Poisson regression	Yes	Yes
Daiet al.	Peripheral blood Morning urine	W: 1.715 C: 497	Male	W: 41 C: 42.48 ± 8.04	MN SNP ELISA Gas chromatography–mass spectrometry HPLC	1000 27	Not reported	Cell count Heparin anticoagulated plasma of BPDE‐Alb Urinary PAH metabolites Oxidative DNA‐damage levels (8‐OHdG)	Yes	No	No	ANOVA Kruskal–Wallis χ^2^ tests	Yes	No
Sureshkumar et al.	Peripheral blood	W: 27 C: 27 ** Both: G1: 12 G2: 15	Male	W: G1: 27.9 ± 3.9 G2: 44.4 ± 5.8 C: G1: 27.5 ± 4.4 G2: 43.5 ± 5.2	MN Comet assay RFLP‐PCR	1000 100 metaphysis	Giemsa	Cell count Cell count Genotypic analysis for XRCC1 399 Arg/Gln polymorphism.	Yes	No	Yes	SPSS ANOVA	Yes	No
Ada et al.	Peripheral blood lymphocytes	W: 50 C: 50	Male	W: 40.4 ± 6.6 years C: 38.7 ± 9.5 years	MN Comet assay Genotyping for polymorphisms HPLC	2000 cells	May–Grünwald Giemsa	CBMN frequency Chromatid breaks, acentric fragments, dicentrics and gaps Genotyped for CYP1A1, CYP1B1, EPHX1, GSTM1, GSTT1 and GSTP1 Urinary 1‐OHP	Yes	Yes	Yes	Student's *t*‐test Mann–Whitney U Spearman correlation Shapiro–Wilk	Yes	No
Huang et al. (2012)	Peripheral blood	W bottom: 67 W side: 57 W top: 78 Cl: 96	Male	W bottom: 38.5 ± 6.6 years W side: 37.4 ± 6.5 years W top: 37 ± 6.3 years C: 37 ± 5 years	Comet assay HPLC Genotyping for polymorphisms	Not reported	Ethidium bromide	Demographic and lifestyle factors TM Plasma BPDE‐Alb Genotyped for CYP1A1, CYP1B1, CYP2B6 and CYP2E1 Air sampling and analysis of airborne PAHs	Yes	No	No	ANOVA χ^2^ tests Kruskal–Wallis	Yes	Yes
Chen et al.	Peripheral blood lymphocytes Urine	W: 289	Male	W side: 44.8 ± 9.5 years W top: 44.5 ± 8.3 years	HPLC Genotyping for polymorphisms (SNPs)	Not specified	Not specified	1‐OHP 8‐OHdG Polymorphism for GNMT Air sampling and analysis of airborne PAHs Demographic and lifestyle factors	Yes	No	No	Student *t*‐test ANOVA Regression model Mann–Whitney U Spearman correlation Chi‐square test	No	No
Wang et al.	Peripheral blood Urine	W: 475	Male	Workers: 38.74 ± 8.59 years	Comet assay HPLC Genotyping for polymorphisms (SNPs)	50 cells per slide on comet assay	Ethidium bromide	Demographic and lifestyle factors 1‐OHP Olive TM Genotyped for ERCC1, CSA, CSB, XPA, XPB, XPC, XPD, XPF, XPG and DDB2 Air sampling and analysis of airborne PAHs	Yes	No	Yes	ANOVA χ^2^ tests Student's *t*‐tests	Yes	Yes
Cheng et al.	Peripheral blood Urine	W: 94 C: 64	Male and female	W: 41.2 ± 6.8 years C: 41.8 ± 4.7 years	MN Comet assay SNP	1000 100	Giemsa for MN Ethidium bromide for CA	Cell count Olive TM Polymorphism analysis of the XRCC1 gene: Arg194Trp (C26304T, rs1799782), Arg280His (G27466A, rs25789), Arg399Gln (G28152A, rs25487) and Gln632Gln (G36189A, rs3547) Demographic and lifestyle factors	Yes	No	Yes	χ^2^ tests Two‐sided two‐sample *t*‐test ANCOVA Logistic regression	Yes	Yes
Yang et al.	Peripheral blood Lung tissue	W: 303 C: 297	Male and female	Workers: 38.8 ± 3.8 years Control: 39.3 ± 8.6 years	Come assay SNP	50	Ethidium bromide	Demographic and lifestyle factors Olive TM Genotyped for FEN1 (c.‐69G>A and c.4150G>T)	Yes	No	Yes	Student's *t*‐tests Logistic regression χ^2^ tests Mann–Whitney U ANCOVA	Yes	Yes
Yang et al.	Peripheral blood	Ws: 251 C: 130	Male	W: 37.1 ± 6.4 years C: 37.1 ± 5.1 years	Come assay HPLC SNPs	50	Ethidium bromide	Demographic and lifestyle factors TL Olive TM Genotyped for HSP70‐1, HSP70‐2, and HSP70‐hom Air sampling and analysis of airborne PAHs	Yes	No	No	Shapiro–Wilk Mann–Whitney U Student's *t*‐tests χ^2^ tests	Yes	No
Qiu et al.	Peripheral blood Urine	W: 166 C: 69	Not reported	W: 35–44	MN Come assay SNPs	1000 100	Giemsa for MN Ethidium bromide for Comet assay	Cell count Olive TM 1‐OHP Polymorphism analysis of the XRCC1, ERCC‐2, mEH3, CYP1A1, and GSTP1 Demographic and lifestyle factors	Yes	No	Yes	Path analysis Regression multivariate χ^2^ tests Student's *t*‐tests	Yes	Yes
Cheng et al.	Peripheral blood Urine	W: 140 C: 66	Not reported	W: 39.1 years	MN SNP	1000	Giemsa	Cell count 1‐OHP Genotyped for ERCC1, ERCC2, ERCC4, ERCC5, and ERCC6 Air sampling and analysis of airborne PAHs Demographic and lifestyle factors	Yes	No	Yes	Mann–Whitney U Spearman χ^2^ tests	Yes	No
Chen et al.	Peripheral blood	W: 240 C: 123	Male	Workers: 37.1 ± 4.9 years Control: 37.1 ± 6.2 years	Comet assay HPLC RFLP‐PCR Multiplex PCR	50	Ethidium bromide	Demographic and lifestyle factors Genotyped for AhR, CYP1A1, GSTM1, and GSTT1 Air sampling and analysis of airborne PAHs	Yes	No	Yes	Student's *t*‐tests χ^2^ tests Mann–Whitney U	Yes	No
Leng et al.	Peripheral blood Urine	W: 141 C: 66	Male and female	W: 39 C: 38.1	MN htSNPs	1000	Not reported	Cell count Analyses of air concentrations of benzene‐soluble matter and particulate‐phase benzo[a]pyrene XRCC1 genotyping and haplotype modeling: Arg194Trp, Arg280His, Arg399Gln, and Gln632Gln Analysis of Tyr113His and His139Arg polymorphisms of mEH gene	Yes	No	Yes	Dunnett–Hsumethod	Yes	No
Leng et al.	Peripheral blood Urine	W: 141 C: 66	Male and female	W: 39 ± 7 years C: 38 ± 8 years	MN HPLC	1000	Giemsa	Demographic and lifestyle factors Urinary PAHs metabolites Cell count Genotyped for CYP1A1, CYP2E1, mEH, GSTM1, GSTT1, GSTP1, NQO1, and NAT2 Air sampling and analysis of airborne PAHs	Yes	No	Yes	Student's *t*‐tests Mann–Whitney U χ^2^ tests Spearman ANCOVA	Yes	No
Wu et al.	Peripheral blood	W: 183	Male	41.2 ± 9.0 years	HPLC ELISA PCR‐RFLP	Not specified	Not specified	1‐OHP 8‐OHdG urinary Polymorphism for GSTM1 and GSTT1 Demographic and lifestyle factors	Yes	No	No	Multiple regression Spearman correlation Student's *t*‐test χ^2^ test	Yes	No
Marczynski et al.	Peripheral blood Urine	W:20 C: 47	Male	W: 37.4 ± 9.5 years C: 38.0 ± 8.9 years	HPLC‐UV/EC HPLC‐DAD HPLC‐FD Comet assay PCR‐RFLP Genotyping for polymorphisms	50	Not specified	Analysis of 8‐oxodGuo in WBC DNA Tail moment 1‐OHP Polymorphism for CYP1A1, GSTM1, GSTT1, and GSTP1 Air sampling and analysis of airborne PAHs Demographic and lifestyle factors	Yes	Yes	Yes	ANOVA Mann–Whitney U Kruskal–Wallis	Yes	No
van Delft et al.	Peripheral blood lymphocytes Urothelial cells	W: 35 C: 37	Male	W: 37–39 years C: 37–40 years	MN Comet assay HPLC‐FD DNA adducts Genotyping for polymorphisms	500 MN 100 cells	Giemsa	1‐OHpyr urine SCE frequency and DNA strand breaks PAH‐DNA adduct Polymorphism for GSTM1 and GSTT1	Yes	No	Yes	Student's *t*‐test Mann–Whitney	Yes	No
Kalina et al	Peripheral blood lymphocytes	W:64 C:34	Male	W: 41 years 27–55) C: 40 years (27–58)	CA SCE HPLC‐FD Cytogenetic monitoring Genotyping for polymorphisms	Not reported	Not reported	Chromosomal aberrations SCE frequency HFC frequency SCE‐H Polymorphism for GSTM1 and NAT2 Air sampling and analysis of airborne PAHs	Yes	No	Yes	Spearman Mann–Whitney U test	Yes	No

Abbreviations: C, control group; CA, chromosomal aberration; CR‐RFLP, real‐time reverse transcription polymerase chain reaction—restriction fragment length polymorphism; HFC, cells with high frequency of sister chromatid exchange (SCE); HPLC, high‐performance liquid chromatography; HPLC‐FD, high‐performance liquid chromatography with fluorescence detection; HPLC/DAD, high‐performance liquid chromatography with diode array detector; HPLC‐UV/EC, high‐performance liquid chromatography with ultraviolet/electrochemical detection; MN, micronucleus assay; PIRA‐PCR, primer‐introduced restriction analysis polymerase chain reaction; SCE, sister chromatid exchange; SCE‐H, heterogeneity index of sister chromatid exchange; SNP, single nucleotide polymorphism; TL, tail length; TM, tail moment; W, workers (exposed group).

Regarding the number of cells evaluated per individual in the micronucleus assays, eight studies evaluated 1000 cells, two evaluated 2000 cells, and one did not report the exact number. All studies used cell count to analyze data (11 out of 11). Moreover, each included study considered inclusion criteria and properly described the statistical tests used for data analysis (Table 3).

### 7.4 Main Results

All included studies reported adverse effects associated with occupational exposure to PAHs in COWs, with alterations detected at genotoxic, molecular, and clinical levels (Table 4). Elevated micronucleus frequency in peripheral blood lymphocytes was a common finding in several studies (Li et al. 2015; Dai et al. 2014; Sureshkumar et al. 2013; Ada et al. 2013; Cheng et al. 2009; Qiu et al. 2007; Cheng et al. 2007; Cheng et al. 2007; Leng et al. 2004; Leng et al. 2005). Additionally, increased CAs and SCEs were reported, indicating further chromosomal damage (Sureshkumar et al. 2013; Ada et al. 2013; Qiu et al. 2007; Marczynski et al. 2002; van Delft et al. 2001; Kalina et al. 1998). Increased tail moment, a marker of DNA strand breaks, was observed in exposed workers as described by (Huang et al. 2012; Chen et al. 2006; Wang et al. 2010; Yang et al. 2009; Yang et al. 2008). DNA adduct formation (BPDE‐DNA and BPDE‐albumin), an early marker of genotoxic exposure, was reported in several studies (Samir et al. 2019; Dai et al. 2014; Huang et al. 2012; Yang et al. 2009). Elevated levels of 8‐OHdG, an indicator of oxidative stress, were also found (Samir et al. 2019; Dai et al. 2014; Wu et al. 2004; Marczynski et al. 2002). Regarding genetic polymorphisms, variants in DNA repair genes (XRCC1, ERCC1, ERCC2, APEX1, XPC, XPA) (Samir et al. 2019; Li et al. 2015; Sureshkumar et al. 2013; Wang et al. 2010; Cheng et al. 2009; Cheng et al. 2007; Leng et al. 2004), xenobiotic metabolism genes (GSTP1, GSTM1, CYP2B6, mEH) (Huang et al. 2012; Qiu et al. 2007; Leng et al. 2004; Kalina et al. 1998), and cellular stress response genes (HSP70‐1, FEN1) (Yang et al. 2009, 2008) were evaluated and associated with genotoxic effects induced by PAHs.

**TABLE 4:** Main findings of studies in chronological order of authors.

Authors	Cytotoxicity	Genotoxicity	Polymorphism results	Observations
Samir et al.	No results	↑ BPDE‐DNA adducts and ↑ 8‐OHdG in exposed workers	XRCC1 polymorphism associated with DNA adduct levels	↑ PAH exposure confirmed via urinary 1‐OHP; DNA adducts linked to XRCC1 genotype
Xiaoliang et al.	No results	↑ MN in exposed workers	↑ BPDE‐Alb in APEX 1 Glu genotypes; ↑ MN with +148Glu allele (mainly in males)	↑ DNA damage association between APEX1 148Glu; ↑ lung cancer risk in smokers with 148Asp or Glu allele
Dai et al.	No results	↑ MN ↑ 8‐OhdG in exposed workers	Association between ↓MN frequency and SNPs 9p21‐rs1333040, 10p14‐rs1663689, 15q25.1rs3813572, rs3813572, CCþ TC and TC genotype, homozygotes (GG) of rs1663689	Exposure groups had higher levels of BPDE‐Alb adducts and most PAH metabolites except 4‐hydroxyphenanthrene; ↑ 8‐OHdG and MN frequency associated with increased environmental PAH levels, adjusting for age, gender, BMI, smoking, and drinking; ↑ BPDE‐Alb, ↑ MN with PAH exposure; ↑ MN in women and older individuals
Sureshkumar et al.	No results	↑ MN and ↑ CA in exposed workers	XRCC1 399Gln: no significant result	↑ MN frequency in smokers; XRCC1 is involved in the efficient repair of DNA single stand breaks
Ada et al.	No results	↑ MN and ↑ CA in exposed workers	No polymorphism analysis	↑ Cytogenetic damage after ≥ 3 months exposure
Huang et al.	No results	↑ TM in exposed workers	↑ BDPE‐Alb in plasm and ↑ TM in GG genotype; ↓ BDPE‐Alb in plasm and ↓TM in GA (CYP2B6) genotype	↑ PAHs B(a)p air in bottom, side and top of oven (especially top of oven); No significant differences of smoking, cigarettes/day or alcohol use
Chen et al.	No results	↑ TM in exposed workers	Association between ↑ TM and AhR Lys554	↑ PAHs B(a)p and BSM air in the coke‐oven working; No significant differences of smoking, cigarettes/day or alcohol use
Wang et al.	No results	↑ TM in exposed workers	Association between ↑ TM and XPC rs2228001; ↓ TM: XPA rs1800975 and XPC rs3731055	↑ PAHs B(a)p air in bottom, side and top of oven (especially top of oven); ↑ 1‐OHP urine in coke‐oven workers; No significant differences of 1‐OHP urine in age, work duration, smoking, cigarettes/day or alcohol use
Cheng et al.	No results	↑ MN in exposed workers	XRCC1 Arg399Gln possibly reduces DRC	↑ 1‐OHP, ↑ basal DNA damage, ↓ DRC in coke‐oven workers; XRCC1399, InOHP, DRC, age indirectly affects MN frequency
Yang et al.	No results	↑ TM in exposed workers	↑ BDPE‐Alb and ↑ TM in ‐69GG abd ‐69GA genotype; FEN1 SNPs (c.‐69G, c4150G>T) associated with ↑ lung cancer risk	↑ PAHs B(a)p and BSM air in the coke‐oven working; No significant differences of smoking, cigarettes/day or alcohol use
Yang et al.	No results	↑ TM in exposed workers	↑ TM associated with HSP70‐1	↑ PAHs B(a)p and BSM air in the coke‐oven working; No significant differences of smoking, cigarettes/day or alcohol use
Qiu et al.	No results	↑ MN and ↑ CA	Association between ↑ MN and GSTP1 variant; ↑ TM: mEH3 variants	↑ 1‐OHP in XRCC1, ERCC2, and XRCC1‐exon6 variant genotypes
Cheng et al.	No results	↑ MN in exposed workers	XRCC1 Arg399Gln reduces DRC	↑ 1‐OHP, ↑ DNA damage, ↓ DRC; MN frequency influenced by multiple factors in coke workers
Cheng et al.	No results	↑ MN in exposed workers	Association between ↑ MN and ERCC1, ERCC6, ERCC2 polymorphisms	↑ PAH in coke‐oven workers; MN ↑ in older workers
Leng et al.	No results	↑ MN in exposed workers	Association between ↑ MN frequency and XRCC1 Arg194Trp polymorphism	↑ 1‐OHP urine in coke‐oven workers; XRCC1 gene in linkage disequilibrium
Leng et al.	No results	↑ MN in exposed workers	Association between ↑ MN and mEH exon 3 Tyr/Tyr, GSTP1 Val105 and GSTM1 null	↑ PAHs B(a)p and BSM air in the coke‐oven working; ↑ Highest levels of both BSM and B(a)p at top of oven; ↑ 1‐OHP urine in coke‐oven workers, current smokers and male than in female workers; ↑ MN frequencies significantly associated, especially in workers ≥ 40 years
Wu et al.	No results	↑ 8‐OHdG levels in workers from topside ovens	No polymorphism analysis	↑ Oxidative damage in topside workers; exposure via 1‐OHP
Marczynski et al.	No results	↑ DNA strand breaks and oxidative lesions in coke oven workers and ↑ 8‐OHdG	No polymorphism analysis	↑ DNA migration; correlated with individual exposure levels (1‐OHP)
van Delft et al.	No results	↑ SCE, ↑ DNA damage in exposed workers	No polymorphism analysis	↑ DNA damage in workers; PAH exposure via urinary 1‐OHP
Kalina et al.	No results	↑ CA, ↑ SCE, ↑ HFC index in exposed workers	GSTM1 and NAT2 genotyping done	↑ Exposure via personal monitoring; ↑ Chromosomal damage

*Note:* **↑ =** increase.

Abbreviations: 1‐OHP = urinary 1‐hydroxypyrene; BDPE = benzo[a]pyrene‐7, 8‐diol‐9, 10‐epoxides; BSM = benzene‐soluble matter; CA = chromosome aberrations; DRC = DNA repair capacity; HFC = cells with high frequency of SCE; htSNPs = high‐throughput single nucleotide polymorphisms; MN = micronucleus; PAHs = polycyclic aromatic hydrocarbons; SCE = sister chromatid exchange; SNP = single nucleotide polymorphism; TM = tail moment.

Exposure biomarkers such as 1‐OHP and airborne PAHs (B[a]P, BSM) were frequently reported in exposed workers (Samir et al. 2019; Dai et al. 2014; Huang et al. 2012; Chen et al. 2006; Wang et al. 2010; Cheng et al. 2009; Yang et al. 2008; Qiu et al. 2007; Cheng et al. 2007; Leng et al. 2004; Leng et al. 2005; Wu et al. 2004; Marczynski et al. 2002; van Delft et al. 2001). Factors such as smoking, age, and exposure duration were also reported as relevant modulators of genotoxic effects (Li et al. 2015; Dai et al. 2014; Sureshkumar et al. 2013; Huang et al. 2012; Cheng et al. 2009; Yang et al. 2009; Yang et al. 2008; Cheng et al. 2007; Leng et al. 2004).

### 7.5 Quality Assessment

The quality assessment is described in Table 5. According to the adopted criteria, a total of 18 studies were classified as Strong, and three (out of 21) were deemed as Moderate. No study was categorized as Weak.

**TABLE 5:** Quality assessment and final rating of the studies.

Author	Number of confounders	Detail	Rating
Samir et al.	0	—	Strong
Xiaoliang et al.	1	—	Strong
Dai et al.	1	—	Strong
Sureshkumar et al.	0	—	Strong
Ada et al.	0	—	Strong
Huang et al.	2	No reported CA number	Moderate
Chen et al.	0	—	Strong
Wang et al.	0	—	Strong
Cheng et al.	0	—	Strong
Yang et al.	0	—	Strong
Yang et al.	1	—	Strong
Qiu et al.	0	—	Strong
Cheng et al.	0	—	Strong
Cheng et al.	1	No control group	Moderate
Chen et al.	0	—	Strong
Leng et al.	0	—	Strong
Leng et al.	0	—	Strong
Wu et al.	0	—	Strong
Marczynski et al.	2	—	Strong
van Delft et al.	0	No minimum amount of evaluated cells	Moderate
Kalina et al.	0	—	Strong

### 7.6 Data Synthesis

Meta‐analysis was performed in six studies. The other studies were removed from the meta‐analysis because the SD data were not shown and there was a need for more groups for proper comparison. The forest plot was used to graphically display effect sizes and 95% CIs. A two‐tailed *p* < 0.05 was used to determine the statistical significance. Heterogeneity was assessed with Cochran's Q test and quantified with the *I* ^2^ index.

**FIGURE 2:**
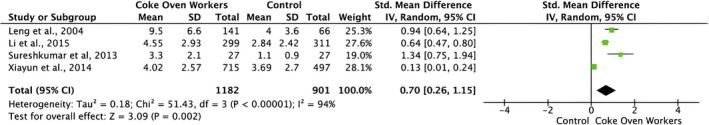
Meta‐analysis from studies using the micronucleus assay.

A meta‐analysis was performed for MN and CA analyses, with different forest plots. The MN studies differed statistically significantly between COWs and Control (SMD = 0.70, 95% CI, 0.26–1.15, *p* = 0.002), with a Tau^2^ = 0.18, Chi^2^ = 51.43, *p*‐value < 0.001, and the *I* ^2^ = 94% (Figure 2).

**FIGURE 3:**
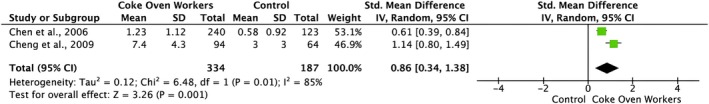
Meta‐analysis from studies using the comet assay.

The outcome of the CA studies differed statistically significantly between COWs and Control (SMD = 0.86, 95% CI, 0.34–1.38, *p* = 0.01), with a Tau^2^ = 0.18, Chi^2^ = 51.43, *p* = 0.001, and the *I* ^2^ = 85% (Figure 3).

For both analyses, according to the meta‐analysis, the “diamond” of the forest graph is shifted to the right side of the null line expressed in the graph. Therefore, the control group differed statistically significantly from the COWs group (*p* < 0.001) and with an increase in micronuclei for the COWs group.

## 8 Discussion

This SR aimed to assess the genotoxic effects of occupational exposure to PAHs in COWs. A total of 21 studies were analyzed, most investigating genotoxic biomarkers such as micronucleus frequency, CAs, SCEs, comet assay tail moment, and oxidative DNA damage indicated by 8‐OHdG.

All studies consistently reported adverse effects associated with PAH exposure, confirming the high genotoxic potential of COEs. Micronuclei frequency was one of the most frequently reported biomarkers, elevated in multiple studies, including those by Leng et al. (2005) and Leng et al. (2004), highlighting chromosomal instability as a common consequence of occupational exposure. Similarly, increased CAs and SCEs were observed, demonstrating that PAHs not only induce DNA strand breaks but also affect the structural integrity of chromosomes. Tail moment analysis in comet assays further revealed extensive DNA strand breaks in peripheral blood lymphocytes, reinforcing the genotoxic profile of PAHs. Oxidative DNA damage, measured by 8‐OHdG, was consistently elevated, suggesting that oxidative stress is a central mechanism mediating PAH‐induced genotoxicity.

The results are in line with previous reports on occupational PAH exposure in other industrial settings. For instance, Baan et al. (2006) highlighted the carcinogenic potential of PAHs and emphasized their genotoxic properties in industrial workers. These studies suggest that the genotoxic effects observed are biologically consistent and likely reflect true occupational risks rather than artifacts of study design. Furthermore, a meta‐analysis was conducted, given that the data mining process was possible for studies with MN and CA. In both analyses, COWs showed genotoxic potential for large effect, despite the high heterogeneity.

Although all studies were able to induce genotoxicity in COWs, differences in exposure assessment methods, population genetics, or study design may interfere with data interpretation with accuracy. Importantly, genetic polymorphisms involved particularly in detoxification genes like glutathione S‐transferase M1 (GSTM1) and T1 (GSTT1) have been proposed as modifiers of individual susceptibility to PAH‐induced DNA damage (Rebbeck 1997). This is because genetic polymorphisms in genes involved in xenobiotic metabolism, DNA repair, and oxidative stress response can significantly influence individual susceptibility to the genotoxic effects of occupational exposure to PAHs (Bolt and Thier 2006). Variants that result in reduced or absent enzymatic activity may impair the body's ability to detoxify reactive metabolites, increasing the risk of DNA damage and cancer development (Kihara et al. 1993; Nazar‐Stewart et al. 1993). Epidemiological studies have suggested that such genetic differences may modulate the biological response to PAHs, particularly in occupational settings; however, findings remain inconsistent across different study populations and designs (Kalina et al. 1998; Pavanello et al. 1999). The observed effects were often modulated by genetic polymorphisms in genes involved in DNA repair, xenobiotic metabolism, and stress response pathways. For instance, variants in XRCC1, ERCC1, ERCC2, APEX1, and XPC were associated with differential susceptibility to DNA damage (Fenech 2007). Polymorphisms in xenobiotic‐metabolizing enzymes, such as GSTM1, GSTP1, mEH, and CYP2B6, were also shown to influence the formation of DNA adducts and the efficiency of detoxification, leading to inter‐individual variability in biomarker levels. Moreover, cellular stress response genes such as HSP70 and FEN1 appeared to play a role in modulating the extent of DNA damage and repair capacity in exposed workers. These findings underscore the importance of considering genetic susceptibility when assessing occupational risk and support the potential utility of personalized biomonitoring strategies in high‐risk environments. On the other hand, the role of genetic susceptibility in modulating this association remains underexplored and is complicated by heterogeneity across studies.

According to established guidelines for genotoxicity assays, the minimum number of cells to be analyzed is 1000 lymphocytes for the micronucleus assay in peripheral blood, while the comet assay requires at least 50 cells per individual (Collins and Dobson 2009). Ensuring compliance with these methodological thresholds is essential for consistent and comparable genotoxicity evaluation in occupational settings. Studies that did not adhere to these standards were flagged during quality assessment, contributing to lower scores, reflecting concerns about the reliability and reproducibility of genotoxicity measurements. Reviewing the included studies, most complied with the lymphocyte standard, and the comet assay was performed according to the minimum cell requirement in all studies which informed the amount of evaluated cells.

Regarding the quality assessment, we understand our findings can be considered reliable since all studies were deemed as either Strong or Moderate. No study was categorized as weak. This means that the information available in the literature within the field is robust and consistent, since all studies do not present relevant bias following the experimental design.

Despite the consistent evidence of genotoxicity, some limitations were identified. Some studies lacked proper cytotoxicity analyses, positive and negative controls, or blinded evaluations, which may introduce bias and affect the reliability of results. Differences in sample size, demographic characteristics, and exposure assessment methods complicate direct comparisons between studies. Furthermore, most studies were cross‐sectional, limiting the ability to infer causal relationships and long‐term health outcomes. Notably, lifestyle factors such as smoking, alcohol consumption, age, and duration of occupational exposure were frequently identified as modifiers of genotoxic outcomes, highlighting the need for comprehensive exposure and confounder assessment.

The evidence supports the role of both environmental exposure and genetic susceptibility in modulating these outcomes. In conclusion, occupational exposure to PAHs in COWs is consistently associated with genotoxic effects, including chromosomal instability, DNA strand breaks, oxidative DNA damage, and the formation of DNA adducts. To improve worker safety and the quality of research, future studies should employ longitudinal designs, standardized biomarker assessments, robust control groups, and blinded analyses. Routine biomonitoring using validated genotoxicity and exposure biomarkers, combined with assessment of genetic susceptibility, may enhance early detection of adverse effects. Employers and regulatory agencies should prioritize engineering controls, effective personal protective equipment, and periodic health surveillance to mitigate PAH exposure and reduce long‐term genotoxic risk among workers.

## Author Contributions

Study design: T.G.P. and D.A.R. Data search: T.G.P., V.F.N., G.A.H.G., R.A.S., and D.A.R. Data analysis: T.G.P., V.F.N., G.A.H.G., R.A.S., and D.A.R. Meta‐analysis: W.M.T. Writing the paper: T.G.P., V.F.N., G.A.H.G., W.M.T., R.A.S., A.C.M.M., L.L.G., and D.A.R.

## Funding

The authors acknowledge research grants received from CNPq (Conselho Nacional de Desenvolvimento Científico e Tecnológico, Grant Number #001) for a productivity fellowship (D.A.R.).

## Ethics Statement

The authors have nothing to report.

## Consent

The authors have nothing to report.

## Conflicts of Interest

The authors declare no conflicts of interest.

## Data Availability

The data that support the findings of this study are available from the corresponding author upon reasonable request.

## References

[jat70098-bib-0001] Ada, A. O. , C. Demiroglu , M. Yilmazer , et al. 2013. “Cytogenetic Damage in Turkish Coke Oven Workers Exposed to Polycyclic Aromatic Hydrocarbons: Association With CYP1A1, CYP1B1, EPHX1, GSTM1, GSTT1, and GSTP1 Gene Polymorphisms.” Archives of Industrial Hygiene and Toxicology 64, no. 3: 359–369. 10.2478/10004-1254-64-2013-2328. 24084344

[jat70098-bib-0004] Baan, R. , Y. Grosse , K. Straif , et al. 2006. “Carcinogenicity of Coal‐Tar Pitch, Benzene, and PAHs in Occupational Settings.” Lancet Oncology 7, no. 12: 1019–1020. 10.1016/S1470-2045(06)71008-2.

[jat70098-bib-0005] Bolt, H. M. , and R. Thier . 2006. “Relevance of the Deletion Polymorphisms of the Glutathione S‐Transferases GSTT1 and GSTM1 in Pharmacology and Toxicology.” Current Drug Metabolism 7, no. 6: 613–628. 10.2174/138920006778226473. 16918316

[jat70098-bib-0006] Bonassi, S. , H. Norppa , M. Ceppi , et al. 2008. “Chromosomal Aberration Frequency in Lymphocytes Predicts the Risk of Cancer: Results From a Pooled Cohort Study of 22,358 Subjects in 11 Countries.” Carcinogenesis 29, no. 6: 1178–1183. 10.1093/carcin/bgn119. 18356148 PMC2443275

[jat70098-bib-0007] Bonassi, S. , A. Znaor , M. Ceppi , et al. 2007. “An Increased Micronucleus Frequency in Peripheral Blood Lymphocytes Predicts the Risk of Cancer in Humans.” Carcinogenesis 28, no. 3: 625–631. 10.1093/carcin/bgl188. 16973674

[jat70098-bib-0010] Chen, Y. , Y. Bai , J. Yuan , et al. 2006. “Association of Polymorphisms in AhR, CYP1A1, GSTM1, and GSTT1 Genes With Levels of DNA Damage in Peripheral Blood Lymphocytes Among Coke‐Oven Workers.” Cancer Epidemiology, Biomarkers & Prevention 15, no. 9: 1703–1707. 10.1158/1055-9965. 16985033

[jat70098-bib-0011] Cheng, J. , S. Leng , Y. Dai , et al. 2007. “Association Between Nucleotide Excision Repair Gene Polymorphisms and Chromosomal Damage in Coke‐Oven Workers.” Biomarkers 12, no. 1: 76–86. 10.1080/13547500600950168. 17438655

[jat70098-bib-0012] Cheng, J. , S. Leng , H. Li , et al. 2009. “Suboptimal DNA Repair Capacity Predisposes Coke‐Oven Workers to Accumulate More Chromosomal Damages in Peripheral Lymphocytes.” Cancer Epidemiology, Biomarkers & Prevention 18, no. 3: 987–993. 10.1158/1055-9965. 19240242

[jat70098-bib-0013] Collins, A. R. , and V. L. Dobson . 2009. “The Comet Assay: What Can It Really Tell Us?” Mutation Research, Fundamental and Molecular Mechanisms of Mutagenesis 681, no. 1: 1–9. 10.1016/j.mrfmmm.2009.02.001.18773969

[jat70098-bib-0014] Dai, X. , S. Deng , T. Wang , et al. 2014. “Associations Between 25 Lung Cancer Risk‐Related SNPs and Polycyclic Aromatic Hydrocarbon‐Induced Genetic Damage in Coke Oven Workers.” Cancer Epidemiology, Biomarkers & Prevention 23, no. 6: 986–996. 10.1158/1055-9965. 24692499

[jat70098-bib-0015] Fenech, M. 2000. “The In Vitro Micronucleus Technique.” Mutation Research 455, no. 1–2: 81–95. 10.1016/S1383-5742(00)00065-7. 11113469

[jat70098-bib-0016] Fenech, M. 2007. “Cytokinesis‐Block Micronucleus Cytome Assay.” Nature Protocols 2, no. 5: 1084–1104. 10.1038/nprot.2007.77. 17546000

[jat70098-bib-0017] Guedes Pinto, T. , T. A. Dias , A. C. M. Renno , M. de Barros Viana , and D. A. Ribeiro . 2024. “The Role of Genetic Polymorphisms for Inducing Genotoxicity in Workers Occupationally Exposed to Benzene: A Systematic Review.” Archives of Toxicology 98, no. 7: 1991–2005. 10.1007/s00204-024-03744-z. 38600397

[jat70098-bib-0018] Hong, Y. C. , E. Y. Park , M. S. Park , et al. 2000. “Community Residents' Exposure to Airborne Polycyclic Aromatic Hydrocarbons and Urinary 1‐Hydroxypyrene Levels.” Science of the Total Environment 263, no. 1–3: 165–174. 10.1016/S0048-9697(00)00631-9.

[jat70098-bib-0019] Huang, G. , H. Guo , and T. Wu . 2012. “Genetic Variations of CYP2B6 Gene Were Associated With Plasma BPDE‐Alb Adducts and DNA Damage Levels in Coke Oven Workers.” Toxicology Letters 211, no. 3: 232–238. 10.1016/j.toxlet.2012.04.004. 22521834

[jat70098-bib-0021] IARC Working Group on the Evaluation of Carcinogenic Risks to Humans . 2012. Personal Habits and Indoor Combustions. Vol. 100E. International Agency for Research on Cancer. PMC4781577 23193840

[jat70098-bib-0022] Kalina, I. , P. Brezáni , D. Gajdosová , et al. 1998. “Cytogenetic Monitoring in Coke Oven Workers.” Mutation Research 1, no. 1: 9–17. 10.1016/s1383-5718(98)00089-8. 9729241

[jat70098-bib-0024] Kihara, M. , A. Hirvonen , and H. Vainio . 1993. “Genetic Polymorphisms in Glutathione S‐Transferases and Susceptibility to Lung Cancer Among Coke Oven Workers.” Carcinogenesis 14, no. 5: 1025–1029. 10.1093/carcin/14.5.1025.

[jat70098-bib-0025] Leng, S. , J. Cheng , L. Zhang , et al. 2005. “The Association of XRCC1 Haplotypes and Chromosomal Damage Levels in Peripheral Blood Lymphocyte Among Coke‐Oven Workers.” Cancer Epidemiology, Biomarkers & Prevention 14, no. 5: 1295–1301. 10.1158/1055-9965. 15894689

[jat70098-bib-0026] Leng, S. , Y. Dai , Y. Niu , et al. 2004. “Effects of Genetic Polymorphisms of Metabolic Enzymes on Cytokinesis‐Block Micronucleus in Peripheral Blood Lymphocyte Among Coke‐Oven Workers.” Cancer Epidemiology, Biomarkers & Prevention 13, no. 10: 1631–1639. 15466980

[jat70098-bib-0027] Li, X. , J. Wei , P. Xu , et al. 2015. “The Interaction of APEX1 Variant With Polycyclic Aromatic Hydrocarbons on Increasing Chromosome Damage and Lung Cancer Risk Among Male Chinese.” Molecular Carcinogenesis 54, no. Suppl 1: E103–E111. 10.1002/mc.22195. 25156607

[jat70098-bib-0028] Malacarne, I. T. , W. M. Takeshita , M. B. Viana , A. C. M. Renno , and D. A. Ribeiro . 2023. “Is Micronucleus Assay a Suitable Method for Biomonitoring Children Exposed to X‐Ray? A Systematic Review With Meta‐Analysis.” International Journal of Radiation Biology 99, no. 10: 1522–1530. 10.1080/09553002.2023.2194405. 36952616

[jat70098-bib-0029] Marczynski, B. , H. P. Rihs , B. Rossbach , et al. 2002. “Analysis of 8‐Oxo‐7,8‐dihydro‐2′‐deoxyguanosine and DNA Strand Breaks in White Blood Cells of Occupationally Exposed Workers: Comparison With Ambient Monitoring, Urinary Metabolites and Enzyme Polymorphisms.” Carcinogenesis 23, no. 2: 273–281. 10.1093/carcin/23.2.273. 11872632

[jat70098-bib-0030] Nazar‐Stewart, V. , J. Brockmoller , and M. Deakin . 1993. “GSTM1 Null Genotype and Lung Cancer Risk Among Coke Oven Workers.” Carcinogenesis 14, no. 5: 1021–1024. 10.1093/carcin/14.5.1021. 7684953

[jat70098-bib-0031] Pavanello, S. , I. Kalina , and A. Mantovani . 1999. “GSTM1 Polymorphisms and Biomarkers of Exposure and Effects in Occupational Settings.” Mutation Research 445, no. 1–2: 137–145. 10.1016/S1383-5742(99)00031-2.

[jat70098-bib-0032] Pinto, T. G. , W. M. Takeshita , A. C. M. Renno , P. R. Cur y , J. J. Dos Santos , and D. A. Ribeiro . 2025. “Is Micronucleus Assay a Useful Marker in Gingiva, Tongue, and Palate for Evaluating Cytogenetic Damage Induced by Chemical, Physical, and Biological Agents In Vivo? A Systematic Review With Meta‐Analysis.” Journal of Applied Toxicology 45, no. 1: 117–134. 10.1002/jat.4662. 38951124

[jat70098-bib-0033] Qiu, L. , S. Leng , Z. Wang , Y. Dai , Y. Zheng , and Z. Wang . 2007. “Path Analysis of Biomarkers of Exposure and Early Biological Effects Among Coke‐Oven Workers Exposed to Polycyclic Aromatic Hydrocarbons.” Cancer Epidemiology, Biomarkers & Prevention 16, no. 6: 1193–1199. 10.1158/1055-9965. 17548684

[jat70098-bib-0034] Rebbeck, T. R. 1997. “Glutathione S‐Transferase Polymorphisms and Cancer Susceptibility.” Cancer Epidemiology, Biomarkers & Prevention 6, no. 9: 733–743. 9298582

[jat70098-bib-0035] Samir, A. M. , D. A. Shaker , M. M. Fathy , et al. 2019. “Urinary and Genetic Biomonitoring of Polycyclic Aromatic Hydrocarbons in Egyptian Coke Oven Workers: Associations Between Exposure, Effect, and Carcinogenic Risk Assessment.” International Journal of Occupational Environmental Medicine 10, no. 3: 124–136. 10.15171/ijoem.2019.1541. 31325295 PMC6708401

[jat70098-bib-0036] Sureshkumar, S. , V. Balachandar , S. M. Devi , et al. 2013. “Estimation of Cytogenetic Risk Among Coke Oven Workers Exposed to Polycyclic Aromatic Hydrocarbons.” Acta Biochimica Polonica 60, no. 3: 375–379. 24040626

[jat70098-bib-0037] van Delft, J. H. , M. S. Steenwinkel , J. G. van Asten , et al. 2001. “Biological Monitoring the Exposure to Polycyclic Aromatic Hydrocarbons of Coke Oven Workers in Relation to Smoking and Genetic Polymorphisms for GSTM1 and GSTT1.” Annals of Occupational Hygiene 45, no. 5: 395–408. 10.1016/S0003-4878(00)00065-X. 11418090

[jat70098-bib-0042] Vimercati, L. , L. Bisceglia , D. Cavone , et al. 2020. “Environmental Monitoring of PAHs Exposure, Biomarkers and Vital Status in Coke Oven Workers.” International Journal of Environmental Research and Public Health 17, no. 7: 2199. 10.3390/ijerph17072199. 32218300 PMC7178092

[jat70098-bib-0038] Wang, F. , Y. He , H. Guo , et al. 2010. “Genetic Variants of Nucleotide Excision Repair Genes Are Associated With DNA Damage in Coke Oven Workers.” Cancer Epidemiology, Biomarkers & Prevention 19, no. 1: 211–218. 10.1158/1055-9965. 20056640

[jat70098-bib-0039] Wu, M.‐T. , C.‐H. Pan , C.‐Y. Chen , et al. 2004. “Lack of Modulating Influence of *GSTM1* and *GSTT1* Polymorphisms on Urinary Biomonitoring Markers in Coke‐Oven Workers.” American Journal of Industrial Medicine 46: 112–119. 10.1002/ajim.20043. 15273962

[jat70098-bib-0040] Yang, M. , H. Guo , C. Wu , et al. 2009. “Functional FEN1 Polymorphisms Are Associated With DNA Damage Levels and Lung Cancer Risk.” Human Mutation 30, no. 9: 1320–1328. 10.1002/humu.21060. 19618370

[jat70098-bib-0041] Yang, X. , J. Yuan , J. Sun , et al. 2008. “Association Between Heat‐Shock Protein 70 Gene Polymorphisms and DNA Damage in Peripheral Blood Lymphocytes Among Coke‐Oven Workers.” Mutation Research 649, no. 1–2: 221–229. 10.1016/j.mrgentox.2007.09.004. 17988935

